# Toward a Unified Definition of the Human Core: The Total Body Core Model

**DOI:** 10.7759/cureus.105599

**Published:** 2026-03-21

**Authors:** Fernando G Paredes

**Affiliations:** 1 Human Movement and Performance, Fusion Fitness Studio, Doylestown, USA

**Keywords:** biomechanics, core stability, functional anatomy, human core, injury prevention, load transfer, movement science, performance training

## Abstract

This technical report proposes a unified, system-level definition of the human core integrating anatomical, biomechanical, and functional perspectives. Despite the central role of core function in spinal stability, movement efficiency, and injury reduction, no comprehensive definition has been consistently adopted across rehabilitation, fitness, and performance domains. Relevant literature on core stability and lumbopelvic-hip complex function is reviewed, and the Total Body Core Model (TBCM) is introduced as an integrative conceptual framework informed by cross-disciplinary research and long-term practitioner observation. The model provides a structured synthesis intended to support scholarly dialogue, guide future investigation, and facilitate empirical evaluation of integrated core function. The TBCM organizes core function into four interdependent layers (deep stabilizing systems, structural stabilizers, myofascial cross-bridges, and peripheral sensory modulators), integrating muscular, fascial, neuromechanical, and proximal-distal interactions within a single framework. The model is proposed as a foundational step toward establishing a standardized operational definition of the human core.

## Introduction

The concept of the “core” is central to rehabilitation, athletic performance, and general fitness, yet it remains inconsistently defined across domains. The term “core” has been described in several ways across rehabilitation, sports medicine, and strength and conditioning literature. Early clinical perspectives emphasized the stabilizing system of the spine, describing coordinated interaction between passive structures, active musculature, and neural control in maintaining spinal stability [1]. Strength and conditioning literature has often defined the core more broadly as the musculature of the trunk responsible for stabilization, posture, and efficient force transmission during movement [2]. In sports medicine, the concept has frequently been expanded to include the lumbopelvic-hip complex as a functional region responsible for transferring forces between the upper and lower extremities during athletic tasks [3]. Rehabilitation research has further highlighted the role of deep trunk musculature, particularly the transversus abdominis and multifidus, in segmental spinal stabilization and motor control [4]. While these perspectives share common elements, they vary considerably in scope and emphasis.

Despite extensive research into spinal stability, lumbopelvic mechanics, and proximal-distal force transfer, substantial variability persists in how core function is conceptualized and operationalized [1-5]. This lack of standardization contributes to fragmented approaches, inconsistent terminology, and variable outcomes. The consequences are practical as well as conceptual. Without a shared framework, rehabilitation professionals lack consistent assessment reference points, strength and conditioning specialists lack unified programming language, and consumers receive conflicting guidance regarding training for spinal support and movement control. This misalignment represents a persistent challenge in movement science. This paper addresses that gap by synthesizing relevant literature and proposing the Total Body Core Model (TBCM) as an integrative framework. Developed through long-term applied practice and informed by anatomical, biomechanical, fascial, and neuromuscular research, the model is intended as a structured synthesis that the field can evaluate and refine. The TBCM conceptualizes the core as a layered, total-body system extending beyond the trunk to include myofascial continuities and distal sensory influences, providing a practical scaffold for improved clarity and cross-disciplinary consistency.

## Technical report

Study design and purpose

This work is presented as a theoretical, integrative framework informed by practitioner observation and guided by anatomical, biomechanical, and neuromuscular research [1,4]. The objective is to describe the development process, governing principles, and evaluation criteria underlying the TBCM. This report does not present experimental data. Instead, it follows a conceptual synthesis approach commonly used in rehabilitation science and applied physiology, integrating empirical research, expert consensus, and long-term clinical observation to address a persistent definitional gap.

Practitioner-informed framework development

The TBCM emerged from more than three decades of clinical work across rehabilitation, general fitness, and performance populations. Recurrent patterns of success and failure were not fully explained by trunk-centric or muscle-isolation models. The framework was developed inductively through observation of movement breakdown and recovery patterns [5], compensatory strategies involving the hips, spine, shoulders, hands, and feet, discrepancies between localized core training and global movement outcomes, and variability in training tolerance and injury recurrence. These observations guided iterative refinement to better reflect real-world stabilization and force-transfer behavior.

Evidence-guided integration

Practitioner observations were continually evaluated against established scientific domains, including spinal stability and motor control [1,2,4], lumbopelvic-hip biomechanics [3], sensory input and motor coordination [6,7], proximal-distal neuromechanical coupling [8-10], myofascial force transmission [11-13], integrated functional training and force transfer principles [14,15], spinal stability modeling and load transfer research [16], and classification of movement-related low back pain disorders [17].

Criteria for model construction

Model development followed explicit criteria: biological normalcy, functional (not purely anatomical) organization, system integration over isolation, applicability across rehabilitation, fitness, and performance settings, and lifespan scalability [5]. Existing models addressed components of core function but not the integrated system.

Framework synthesis process

Core-related structures were grouped by primary roles in stabilization, force transfer [14,15], and sensory amplification, consistent with established spinal stability models [16]. This process yielded four interdependent functional layers. The layers are not sequential training stages but concurrently active systems whose relative contribution varies by task demands and individual readiness. The structure was refined iteratively to maintain internal coherence.

Positioning of the model

The TBCM is proposed as an organizing framework rather than a definitive solution. By unifying previously disconnected components of core function, it provides a practitioner-informed, evidence-guided structure intended for scholarly evaluation and future testing.

Framework overview - the Total Body Core Model framework

The TBCM organizes core function into four interdependent layers defined by functional contribution to whole-body stabilization and force transfer rather than isolated anatomy. Core function emerges from the coordinated interaction of muscular, fascial, neurological, and sensory systems. Dysfunction in any layer may impair movement efficiency, load tolerance, or resilience.

Layer 1: Deep Core Stabilizers (Foundational Control)

This layer includes deep segmental stabilizers responsible for intra-abdominal pressure regulation, segmental spinal control, and postural reflexes. Primary contributors include the diaphragm, transversus abdominis, multifidus, and pelvic floor musculature. These muscles function through low-threshold tonic activation and coordinated pressure regulation, increasing spinal stiffness and stabilizing individual vertebral segments during movement. Activation of these stabilizers typically occurs in a feed-forward manner prior to limb motion, helping prepare the spine for load and maintain segmental alignment during dynamic tasks. When functioning effectively, this system supports efficient load distribution and movement readiness rather than high levels of force production. Deficits within this stabilizing system have been associated with impaired segmental control, delayed stabilization responses, and reduced load tolerance. Integrity of this layer is therefore necessary, though not sufficient, for full core function within the integrated system [1,4].

Layer 2: Structural Core (Lumbopelvic-Hip Complex)

This layer represents the structural core of the trunk and pelvis, responsible for generating gross trunk stiffness, maintaining pelvic orientation, and transferring load between the upper and lower body. Key contributors include the abdominal wall (rectus abdominis, internal and external obliques), spinal extensors, quadratus lumborum, and the musculature of the hips and pelvis, including the gluteal complex. These muscles function through coordinated activation to control trunk motion, resist excessive spinal movement, and stabilize the pelvis during dynamic tasks. By regulating trunk stiffness and pelvic alignment, the lumbopelvic-hip complex enables efficient force transfer between the upper and lower extremities during locomotion, lifting, and athletic movement. Compromise within this system commonly presents as excessive spinal motion, pelvic instability, and compensatory activation of superficial musculature. Effective performance of this layer depends on coordination with both deep stabilizing systems and distal neuromechanical contributors within the broader core network [2,3].

Layer 3: Myofascial Anchors (Upper and Lower Cross-Bridges)

This layer represents the myofascial continuities that integrate the trunk with the upper and lower extremities, enabling diagonal force transmission, rotational control, and distributed load sharing across the body. Key structures include the thoracolumbar fascia and interconnected muscular-fascial sling systems that link the shoulders, trunk, pelvis, and hips. Through these connective tissue networks, forces generated in one region of the body can be transmitted across multiple segments, contributing to coordinated movement patterns and rotational power generation. These cross-body connections support efficient transfer of mechanical energy during activities such as gait, throwing, and lifting. Disruption within these myofascial linkages may contribute to reduced rotational efficiency, asymmetrical loading patterns, and increased compensatory demand on the spine. Inclusion of this layer distinguishes the TBCM from trunk-centric models by emphasizing the role of fascial force transmission and cross-body integration within core function [10-12].

Layer 4: Peripheral Sensory and Neuromechanical Modulators

The fourth functional layer of the TBCM describes peripheral sensory and neuromechanical systems capable of influencing proximal stabilization. These structures are not proposed as structural components of the core itself, but rather as distal contributors that may modulate trunk activation through sensory feedback, neuromechanical coupling, and pressure-based stabilization mechanisms. Distal sensory structures such as the hands and feet provide dense afferent input that contributes to postural regulation and movement coordination. Mechanoreceptors within the plantar surface of the foot play a critical role in balance and postural control, influencing proximal muscle recruitment and stabilization strategies during functional tasks [18]. Similarly, hand and grip function demonstrate functional coupling with trunk stability, as grip strength varies with trunk position and stabilization demands [19]. Grip strength has also been shown to correlate with overall neuromuscular capacity and physical health across populations [20]. Respiratory mechanics represent another important contributor to trunk stabilization. Coordination between respiratory musculature, intra-abdominal pressure, and trunk musculature plays a significant role in spinal stiffness and load tolerance [16]. These interactions illustrate how breathing mechanics can influence trunk stabilization during functional movement. Together, these findings support the concept that distal sensory inputs and respiratory pressure systems may influence proximal stabilization through sensorimotor integration and neuromechanical coupling. Within the TBCM framework, these mechanisms are described as peripheral modulators capable of amplifying or constraining stabilization strategies depending on task demands and neuromuscular coordination.

To enhance conceptual clarity, the four functional layers of the TBCM are summarized in Table 1.

**Table 1:** Functional layers of the Total Body Core Model (TBCM).

Layer	Functional role	Primary components	Clinical/performance relevance	Key supporting evidence
Layer 1: Deep core stabilizers	Foundational segmental control and intra-abdominal pressure regulation	Diaphragm, transversus abdominis, multifidus, and pelvic floor	Provides anticipatory spinal stability and postural control during limb movement	Hodges & Richardson [4]; Panjabi [1]
Layer 2: Structural core (lumbopelvic–hip complex)	Global trunk stiffness and load transfer between the upper and lower body	Abdominal wall, spinal extensors, hip stabilizers, and pelvic musculature	Supports force generation, anti-rotation control, and load tolerance	Kibler et al. [3]; McGill [2]
Layer 3: Myofascial anchors	Diagonal force transmission and elastic energy transfer	Thoracolumbar fascia, anterior/posterior sling systems, and myofascial continuities	Enables rotational power, cross-body coordination, and distributed load sharing	Schleip et al. [10]; Wilke et al. [11]; Vleeming et al. [12]
Layer 4: Peripheral sensors and amplifiers	Sensory modulation and neuromechanical amplification of proximal stability	Plantar mechanoreceptors, grip musculature, and respiratory pressure systems	Influences proximal stability through afferent input and environmental interaction	Zazulak et al. [9]; Bernstein [6]

Interdependence of the layers

A defining feature of the TBCM is the concurrent activity of all four layers, with their relative contributions varying according to task demands, fatigue, injury history, and individual capacity. Rather than functioning independently, these systems interact dynamically to regulate stabilization, load transfer, and movement coordination. Dysfunction in any layer may alter the mechanical and neuromuscular demands placed on the others. For example, deficits in deep stabilizers may increase reliance on structural musculature for spinal stiffness, inefficiencies in myofascial force transmission may elevate segmental loading on the spine, and impaired distal sensory input may disrupt proximal motor control and coordination. Recognizing these interactions may support more precise assessment and intervention strategies within rehabilitation and performance settings.

Summary of framework results

The TBCM provides an integrated organizational framework for core functions that extend beyond the trunk while maintaining definitional clarity. By incorporating muscular, fascial, neurological, and sensory domains, the model offers a unified structure applicable across rehabilitation, fitness, and performance contexts.

## Discussion

Reframing the core as a total-body system

The findings highlight a central issue in contemporary movement science: despite broad agreement that the core is essential to spinal stability, force transfer, and injury reduction [1-3,10], the field lacks a unified structural framework explaining how these functions are distributed throughout the body. The TBCM addresses this gap by organizing anatomical, biomechanical, and neuromechanical evidence into a layered integrative system extending beyond the trunk to include myofascial continuities and distal sensory contributors. The TBCM does not contradict existing models of core stability but contextualizes and expands them. Deep stabilizers, lumbopelvic control, and anti-movement strategies remain essential. However, when these elements are treated in isolation, without consideration of fascial force transmission or distal sensory input, training and rehabilitation approaches may be incomplete or inconsistently effective.

Integration across disciplines

A primary strength of the TBCM is its ability to reconcile perspectives often treated as competing. Clinical models emphasize segmental stability and load tolerance, performance models prioritize force transfer and power generation, and movement education systems focus on coordination and adaptability. The layered structure allows these priorities to coexist within a single framework. By incorporating myofascial continuities and proximal-distal interactions, the model aligns with emerging fascia science while preserving established musculoskeletal and neuromechanical principles [11-13]. This integration reflects growing recognition that human movement cannot be fully explained by isolated muscle action or by fascial systems considered independently of neuromuscular control.

Clarifying a persistent blind spot

The absence of a standardized core definition has practical consequences. Without a shared framework, clinicians and coaches rely on fragmented terminology and discipline-specific assumptions that do not translate consistently across contexts. This contributes to variable outcomes, conflicting recommendations, and professional and consumer confusion [3,17]. The TBCM reframes the issue as a limitation in synthesis rather than research volume. Relevant evidence already exists across anatomy, biomechanics, rehabilitation science, fascia research, and motor control literature; what has been lacking is an organizing structure capable of integrating these domains.

Practitioner-informed perspective

The TBCM is practitioner-informed and grounded in peer-reviewed research and expert consensus. Its structure was refined through long-term observation across diverse populations, offering a complementary perspective to controlled laboratory research [17]. Many accepted movement frameworks followed similar trajectories, emerging from practical necessity before formal validation. The TBCM is positioned as a structured hypothesis intended to invite empirical evaluation.

Implications for research and practice

A layered, total-body definition of the core has implications for both research and application. For researchers, the model offers a scaffold for clearer operational definitions and cross-study comparison. For clinicians and coaches, it provides a conceptual map to guide assessment and progression without prescribing specific techniques. Core function is not localized to a single region or exercise category but emerges from coordinated interaction across muscular, fascial, neurological, and sensory systems [6,7,14], with relative contributions shifting according to task demands and individual capacity.

Toward dialogue, not dogma

The TBCM is proposed as a structured starting point demonstrating that a comprehensive core definition is feasible. By articulating the core as a layered total-body system, the model aims to promote constructive interdisciplinary dialogue. Future research may validate, refine, or challenge elements of the framework. Such outcomes would represent progress by advancing understanding of a fundamental yet inconsistently defined concept in human movement science.

Precedent models and the acceptance of incomplete frameworks

Progress in physiology and movement science often involves the adoption of simplified models that improve communication and application before full biological resolution. These frameworks function as organizational tools that enable advancement while empirical refinement continues.

The sliding filament theory of muscle contraction

The sliding filament theory remains foundational in muscle physiology. Although later research expanded its mechanistic detail, the model was widely adopted because it unified observations across physiology, anatomy, and biomechanics and provided a testable explanatory framework [21].

Heart rate models and the 220 − age formula

The maximal heart rate formula (220 − age) illustrates pragmatic model adoption. Despite known variability and a limited experimental origin, it continues to be widely used because it provides a functional reference point for exercise prescription and population comparisons [22].

Heart rate zone training systems

Heart rate zone frameworks similarly divide cardiovascular intensity into practical training categories. Although physiological thresholds vary between individuals, these systems remain useful for guiding training intensity and clinical exercise prescription [23].

The joint-by-joint approach

In movement science, the joint-by-joint approach proposes alternating mobility and stability roles along the kinetic chain. While not universally applicable, the model has gained traction because it provides a practical framework for movement assessment and programming [24].

The gradual acceptance of fascia as a functional system

Fascia was historically viewed as passive tissue, but is now recognized as a dynamic load-transmitting and sensory system [11,12]. Its delayed acceptance illustrates the challenge of integrating new insights into established paradigms.

Implications for core definition

These examples illustrate how simplified conceptual frameworks often precede complete biological explanation. Within this context, the TBCM is proposed as a similar organizing structure intended to clarify an area of movement science where terminology and conceptual boundaries remain inconsistent.

Framing progress, not finality

The central question is not whether a core model can perfectly capture biological complexity, but whether the absence of such a framework continues to hinder progress. Structured models, when clearly defined and open to refinement, serve as catalysts for advancement. Within this context, the TBCM is offered to advance the conversation rather than conclude it.

To further contextualize the proposed framework within existing literature, Table 2 compares the TBCM with commonly referenced core conceptualizations.

**Table 2:** Comparison of common core conceptualizations vs. TBCM. TBCM: Total Body Core Model; LPHP: lumbopelvic-hip complex.

Conceptual approach	Primary focus	Strengths	Common limitations	How TBCM expands the framework
Deep stabilization models	Segmental spinal control and intra-abdominal pressure	Strong evidence for anticipatory stabilization and motor control	Often trunk-centric and limited integration with global movement	Retains deep stabilizers within a multi-layer system
Lumbopelvic–hip complex models	Structural trunk and pelvic strength	Effective for load tolerance and gross stability	May underrepresent fascial continuity and distal influence	Positions the LPHP as one structural layer within a whole-body system
Functional performance models	Force transfer and athletic movement	Emphasizes power coordination and kinetic chains	Core definitions often implicit or variable	Provides explicit structural organization for force transfer mechanisms
Fascial continuity perspectives	Myofascial force transmission across the body	Highlights the connective tissue's role in movement integration	Sometimes lacks integration with the motor control hierarchy	Incorporates fascial networks alongside muscular and neural systems
Total Body Core Model (TBCM)	Layered integration of stabilizing structural fascial and sensory systems	Offers a unified cross-disciplinary framework	Requires future empirical validation	Serves as an organizing model for research assessment and application

This comparison further highlights the need for an integrative framework capable of reconciling these perspectives.

Limitations

The TBCM is presented as a conceptual and integrative framework rather than an experimentally validated theory. Although grounded in established anatomical, biomechanical, neuromuscular, and fascial research, the model has not yet been tested in prospective trials designed to evaluate its structure or clinical superiority. Accordingly, it should be interpreted as a hypothesis-generating framework intended to guide inquiry and application. The practitioner-informed origin of the TBCM represents both a strength and a limitation. The model was developed through more than three decades of hands-on coaching, assessment, and rehabilitation across diverse adult populations. While this provides ecological validity, it also introduces variability related to population differences, training environments, and observational conditions. Empirical investigation is therefore required to establish broader generalizability.

In addition, the layered structure is designed to organize functionally relevant systems rather than exhaustively catalogue all contributors to core behavior. Layer boundaries are conceptual, and interactions among systems are continuous and task-dependent. As with other accepted frameworks in movement science, simplification is used to improve clarity while acknowledging biological complexity. The TBCM also does not prescribe specific exercises or standardized clinical protocols. Rather, the model is intended to provide an organizing framework to inform assessment, reasoning, and intervention design across rehabilitation, fitness, and performance contexts. Because movement demands, injury history, and population characteristics vary widely, application of the framework necessarily requires practitioner judgment. Future research may explore how the model can inform structured assessment strategies or targeted intervention protocols. Finally, this report does not seek to resolve all debates surrounding core stability, motor control, or fascial mechanics. Instead, it highlights the absence of a unified organizing definition and proposes a structured framework for ongoing evaluation, refinement, and research. Future investigation may evaluate the reliability, validity, and clinical utility of the TBCM across rehabilitation, athletic, and general population settings.

## Conclusions

Despite decades of research on spinal stability and lumbopelvic mechanics, the human core remains inconsistently defined across clinical, fitness, and performance domains. The TBCM proposes a layered, system-level framework integrating deep stabilizers, structural support systems, myofascial force transmission, and distal sensory influences within a unified conceptual structure. By organizing these interacting systems into a single framework, the TBCM aims to improve conceptual clarity and facilitate more consistent communication across rehabilitation, sports science, and performance training disciplines. Rather than serving as a definitive solution, the model is intended to support interdisciplinary dialogue and provide a foundation for future empirical investigation of integrated core function.
